# Supplementary material for: An investigation of the performance of parametric functional forms for the Lorenz curve
Source: PLoS One. 2023 Jun 23;18(6):e0287546. doi: 10.1371/journal.pone.0287546 (PMC10289384; doi:10.1371/journal.pone.0287546)
Supplement: S1 Appendix — (DOCX) [file pone.0287546.s001.docx]

**Appendix**

**Table A1. The values of parameters estimated based on Paul and Shankar [3]’s functional form and the other existing widely used single-parameter functional forms as well as the functional form proposed by Sitthiyot and Holasut [2] using the income data of 20 OECD countries.**

| **Country** | **Year** | **Observed Gini index** | **Estimated parameter** | | | | | | |
| --- | --- | --- | --- | --- | --- | --- | --- | --- | --- |
|  |  |  | **PS** | **KP** | **A** | **C** | **Pareto** | **SH** | |
|  |  |  | $\gamma$ | $\delta$ | $\theta$ | $k$ | $\alpha$ | $a$ | $b$ |
| Slovakia | 2017 | 0.2320 | 0.0000001 | 0.8147 | 0.1680 | 1.3856 | 1.5545 | 0.3971 | 1.5974 |
| Slovenia | 2017 | 0.2370 | 0.0000001 | 0.8473 | 0.1739 | 1.4346 | 1.5808 | 0.4639 | 1.6221 |
| Czech Republic | 2017 | 0.2450 | 0.0000001 | 0.8798 | 0.1804 | 1.4840 | 1.6146 | 0.6009 | 1.6473 |
| Finland | 2017 | 0.2530 | 0.0000001 | 0.9183 | 0.1865 | 1.5396 | 1.6407 | 0.5800 | 1.6772 |
| Belgium | 2017 | 0.2600 | 0.0000001 | 0.9513 | 0.1908 | 1.5852 | 1.6536 | 0.4484 | 1.7034 |
| Norway | 2017 | 0.2610 | 0.0000001 | 0.9415 | 0.1899 | 1.5722 | 1.6549 | 0.5214 | 1.6965 |
| Netherlands | 2017 | 0.2710 | 0.0000001 | 0.9930 | 0.1982 | 1.6458 | 1.6912 | 0.5302 | 1.7361 |
| Denmark | 2017 | 0.2760 | 0.0000001 | 1.0019 | 0.1999 | 1.6588 | 1.7022 | 0.5649 | 1.7438 |
| Austria | 2017 | 0.2790 | 0.0000001 | 1.0335 | 0.2043 | 1.7025 | 1.7187 | 0.5028 | 1.7684 |
| Sweden | 2017 | 0.2800 | 0.0000001 | 1.0322 | 0.2040 | 1.7005 | 1.7171 | 0.4928 | 1.7676 |
| Hungary | 2017 | 0.2810 | 0.0000001 | 1.0469 | 0.2064 | 1.7213 | 1.7280 | 0.5052 | 1.7786 |
| Germany | 2017 | 0.2910 | 0.0000001 | 1.0941 | 0.2137 | 1.7872 | 1.7641 | 0.5236 | 1.8160 |
| Poland | 2017 | 0.2920 | 0.0000001 | 1.0991 | 0.2146 | 1.7944 | 1.7690 | 0.5347 | 1.8199 |
| France | 2017 | 0.2930 | 0.0000001 | 1.0942 | 0.2148 | 1.7891 | 1.7754 | 0.6223 | 1.8165 |
| Switzerland | 2017 | 0.3010 | 0.0000001 | 1.1417 | 0.2215 | 1.8537 | 1.8055 | 0.5805 | 1.8539 |
| Ireland | 2017 | 0.3060 | 0.0000001 | 1.1807 | 0.2270 | 1.9067 | 1.8299 | 0.5604 | 1.8841 |
| Canada | 2017 | 0.3090 | 0.0000001 | 1.1937 | 0.2277 | 1.9224 | 1.8285 | 0.4488 | 1.8958 |
| Luxembourg | 2017 | 0.3090 | 0.0000001 | 1.1990 | 0.2288 | 1.9303 | 1.8359 | 0.4872 | 1.8995 |
| Estonia | 2017 | 0.3160 | 0.0000001 | 1.2433 | 0.2341 | 1.9882 | 1.8566 | 0.3955 | 1.9355 |
| Italy | 2017 | 0.3270 | 0.0000001 | 1.2775 | 0.2395 | 2.0346 | 1.8898 | 0.4382 | 1.9636 |

PS, Paul and Shankar [3]; KP, Kakwani and Podder [5]; A, Aggarwal [9]; C, Chotikapanich [16]; Pareto, Functional form implied by Pareto distribution; SH, Sitthiyot and Holasut [2].

**Table A2. The values of parameters estimated based on Paul and Shankar [3]’s functional form and the other existing widely used single-parameter functional forms as well as the functional form proposed by Sitthiyot and Holasut [2] using the income data of the other 20 countries.**

| **Country** | **Year** | **Income share of the top 20%** | **Estimated parameter** | | | | | | |
| --- | --- | --- | --- | --- | --- | --- | --- | --- | --- |
|  |  |  | **PS** | **KP** | **A** | **C** | **Pareto** | **SH** | |
|  |  |  | $\gamma$ | $\delta$ | $\theta$ | $k$ | $\alpha$ | $a$ | $b$ |
| Thailand | 2017 | 51.12 | 0.3202 | 2.1398 | 0.3434 | 3.1035 | 2.5652 | 0.6113 | 2.6686 |
| Malawi | 2017 | 51.67 | 0.2748 | 2.0508 | 0.3381 | 3.0030 | 2.5370 | 0.8016 | 2.5874 |
| Ecuador | 2015 | 51.90 | 0.3759 | 2.2089 | 0.3503 | 3.1833 | 2.6197 | 0.6174 | 2.7255 |
| Benin | 2015 | 52.13 | 0.4340 | 2.2953 | 0.3586 | 3.2827 | 2.6935 | 0.5923 | 2.8036 |
| Philippines | 2012 | 52.68 | 0.4143 | 2.2532 | 0.3546 | 3.2340 | 2.6534 | 0.6332 | 2.7597 |
| Sri Lanka | 2010 | 54.40 | 0.5525 | 2.4359 | 0.3727 | 3.4418 | 2.8154 | 0.6746 | 2.9134 |
| Nicaragua | 2014 | 54.50 | 0.5695 | 2.4653 | 0.3747 | 3.4745 | 2.8318 | 0.6459 | 2.9397 |
| Bangladesh | 2016 | 54.75 | 0.5695 | 2.4653 | 0.3747 | 3.4745 | 2.8318 | 0.7263 | 2.9313 |
| Costa Rica | 2016 | 54.90 | 0.6142 | 2.5408 | 0.3794 | 3.5572 | 2.8667 | 0.5704 | 3.0066 |
| Saint Lucia | 2016 | 55.39 | 0.6680 | 2.6257 | 0.3863 | 3.6514 | 2.9332 | 0.5480 | 3.0815 |
| Paraguay | 2016 | 55.41 | 0.6639 | 2.6105 | 0.3858 | 3.6347 | 2.9301 | 0.5906 | 3.0648 |
| Mexico | 2016 | 55.70 | 0.6410 | 2.5575 | 0.3832 | 3.5767 | 2.9078 | 0.6823 | 3.0113 |
| India | 2012 | 56.38 | 0.7166 | 2.6837 | 0.3920 | 3.7149 | 2.9887 | 0.6088 | 3.1230 |
| Eswatini | 2010 | 56.73 | 0.7382 | 2.7149 | 0.3943 | 3.7485 | 3.0087 | 0.6073 | 3.1479 |
| Colombia | 2015 | 56.80 | 0.7711 | 2.7691 | 0.3988 | 3.8083 | 3.0576 | 0.6016 | 3.1969 |
| Brazil | 2018 | 58.60 | 0.8784 | 2.9303 | 0.4119 | 3.9823 | 3.1939 | 0.6362 | 3.3279 |
| Egypt | 2013 | 58.78 | 0.8854 | 2.9290 | 0.4129 | 3.9807 | 3.2057 | 0.6917 | 3.3196 |
| Mozambique | 2015 | 59.53 | 0.9134 | 2.9572 | 0.4162 | 4.0101 | 3.2401 | 0.7599 | 3.3314 |
| Honduras | 2012 | 60.10 | 0.9763 | 3.0767 | 0.4234 | 4.1373 | 3.3190 | 0.6923 | 3.4399 |
| Namibia | 2016 | 63.74 | 1.2904 | 3.6505 | 0.4584 | 4.7346 | 3.7517 | 0.6163 | 3.9296 |

PS, Paul and Shankar [3]; KP, Kakwani and Podder [5]; A, Aggarwal [9]; C, Chotikapanich [16]; Pareto, Functional form implied by Pareto distribution; SH, Sitthiyot and Holasut [2].
