# Supplementary material for: An investigation of the performance of parametric functional forms for the Lorenz curve
Source: PLoS One. 2023 Jun 23;18(6):e0287546. doi: 10.1371/journal.pone.0287546 (PMC10289384; doi:10.1371/journal.pone.0287546)
Supplement: Attachment — Submitted filename: PONE-D-22-23738.docx [file pone.0287546.s002.docx]

PLOS ONE

# An investigation of the performance of parametric functional forms for the Lorenz curve

--Manuscript Draft--

| **Manuscript Number:** | PONE-D-22-23738 |
| --- | --- |
| **Article Type:** | Research Article |
| **Full Title:** | An investigation of the performance of parametric functional forms for the Lorenz curve |
| **Short Title:** | Parametric functional forms for estimating the Lorenz curve |
| **Corresponding Author:** | Dr. Thitithep Sitthiyot, Ph.D. Chulalongkorn University Bangkok, THAILAND |
| **Keywords:** | Gini index; Income distribution; Inequality; Lorenz curve; Parametric functional form; Size distribution |
| **Abstract:** | Given that an excellent performance of any parametric functional form for the Lorenz curve that is based on a single country case study and a limited range of distribution must be treated with great caution, this study investigates the performance of a single- parameter functional form proposed by Paul and Shankar (2020) who use income data of Australia to show that their functional form is superior to the other existing widely used functional forms considered in their study. By using both mathematical proof and empirical data of 40 countries around the world, this study demonstrates that Paul and Shankar (2020)’s functional form not only fails to fit the actual observations well but also is generally outperformed by the other popular functional forms considered in their study. Moreover, to overcome the limitation of the performance of a single-parameter functional form on the criterion of the estimated Gini index, this study employs a functional form that has more than one parameter in order to show that, by and large, it performs better than all popular single-parameter functional forms considered in Paul and Shankar (2020)’s study. Thus, before applying any functional form to estimate the Lorenz curve, policymakers should check if it could describe the shape of income distributions of different countries through the changes in parameter values and yield the values of the estimated Gini index that are close to their observed data. Using a functional form that does not fit the actual observations could adversely affect inequality measures and income distribution policies. |
| **Order of Authors:** | Dr. Thitithep Sitthiyot, Ph.D. |
|  | Dr. Kanyarat Holasut, Ph.D. |
| **Additional Information:** |  |
| **Question** | **Response** |
| **Financial Disclosure**  Enter a financial disclosure statement that describes the sources of funding for the work included in this submission. Review the [submission guidelines](http://journals.plos.org/plosone/s/submission-guidelines#loc-financial-disclosure-statement) for detailed requirements. View published research articles from [*PLOS ONE*](http://journals.plos.org/plosone/) for specific examples.  This statement is required for submission and **will appear in the published article** if the submission is accepted. Please make sure it is accurate. | The authors received no specific funding for this work. |

*Powered by Editorial Manager® and ProduXion Manager® from Aries Systems Corporation*

| **Unfunded studies**  Enter: *The author(s) received no specific funding for this work.*  **Funded studies**  Enter a statement with the following details:   - Initials of the authors who received each award - Grant numbers awarded to each author - The full name of each funder - URL of each funder website - Did the sponsors or funders play any role in the study design, data collection and analysis, decision to publish, or preparation of the manuscript? - **NO** - Include this sentence at the end of your statement: *The funders had no role in study design, data collection and analysis, decision to publish, or preparation of the manuscript.* - **YES** - Specify the role(s) played. |  |
| --- | --- |
| * typeset |  |
| **Competing Interests**  Use the instructions below to enter a competing interest statement for this submission. On behalf of all authors, disclose any [competing interests](http://journals.plos.org/plosone/s/competing-interests) that could be perceived to bias this work—acknowledging all financial support and any other relevant financial or non- financial competing interests.  This statement is required for submission and **will appear in the published article** if the submission is accepted. Please make sure it is accurate and that any funding sources listed in your Funding Information later in the submission form are also declared in your Financial Disclosure statement.  View published research articles from  [*PLOS ONE*](http://journals.plos.org/plosone/) for specific examples. | I have read the journal's policy and the authors of this manuscript have the following competing interests:  The findings reported in this manuscript contradict the results previously published in Paul and Shankar (2020). |

*Powered by Editorial Manager® and ProduXion Manager® from Aries Systems Corporation*

| **NO authors have competing interests**  Enter: *The authors have declared that no competing interests exist.*  **Authors with competing interests**  Enter competing interest details beginning with this statement:  *I have read the journal's policy and the authors of this manuscript have the following competing interests: [insert competing interests here]* |  |
| --- | --- |
| * typeset |  |
| **Ethics Statement**  Enter an ethics statement for this submission. This statement is required if the study involved:   - Human participants - Human specimens or tissue - Vertebrate animals or cephalopods - Vertebrate embryos or tissues - Field research   Write "N/A" if the submission does not require an ethics statement.  General guidance is provided below. Consult the [submission guidelines](http://journals.plos.org/plosone/s/submission-guidelines#loc-guidelines-for-specific-study-types) for detailed instructions. **Make sure that all information entered here is included in the Methods section of the manuscript.** | N/A |

*Powered by Editorial Manager® and ProduXion Manager® from Aries Systems Corporation*

| **Format for specific study types**  **Human Subject Research (involving human participants and/or tissue)**   - Give the name of the institutional review board or ethics committee that approved the study - Include the approval number and/or a statement indicating approval of this research - Indicate the form of consent obtained (written/oral) or the reason that consent was not obtained (e.g. the data were analyzed anonymously)   **Animal Research (involving vertebrate animals, embryos or tissues)**   - Provide the name of the Institutional Animal Care and Use Committee (IACUC) or other relevant ethics board that reviewed the study protocol, and indicate whether they approved this research or granted a formal waiver of ethical approval - Include an approval number if one was obtained - If the study involved *non-human primates*, add *additional details* about animal welfare and steps taken to ameliorate suffering - If anesthesia, euthanasia, or any kind of animal sacrifice is part of the study, include briefly which substances and/or methods were applied   **Field Research**  Include the following details if this study involves the collection of plant, animal, or other materials from a natural setting:   - Field permit number - Name of the institution or relevant body that granted permission |  |
| --- | --- |
|  |  |
| **Data Availability**  Authors are required to make all data underlying the findings described fully available, without restriction, and from the time of publication. PLOS allows rare exceptions to address legal and ethical concerns. See the [PLOS Data Policy](http://journals.plos.org/plosone/s/data-availability) and [FAQ](http://journals.plos.org/plosone/s/data-availability#loc-faqs-for-data-policy) for detailed information. | Yes - all data are fully available without restriction |

*Powered by Editorial Manager® and ProduXion Manager® from Aries Systems Corporation*

| A Data Availability Statement describing where the data can be found is required at submission. Your answers to this question constitute the Data Availability Statement and **will be published in the article**, if accepted. |  |
| --- | --- |
| **Important:** Stating ‘data available on request from the author’ is not sufficient. If your data are only available upon request, select ‘No’ for the first question and explain your exceptional situation in the text box. |  |
| Do the authors confirm that all data underlying the findings described in their manuscript are fully available without restriction? |  |
| **Describe where the data may be found in full sentences. If you are copying our sample text, replace any instances of XXX with the appropriate details.**   - If the data are **held or will be held in a public repository**, include URLs, accession numbers or DOIs. If this information will only be available after acceptance, indicate this by ticking the box below. For example: *All XXX files are available from the XXX database (accession number(s) XXX, XXX.*). - If the data are all contained **within the manuscript and/or Supporting Information files**, enter the following: *All relevant data are within the manuscript and its Supporting Information files.* - If neither of these applies but you are able to provide **details of access elsewhere**, with or without limitations, please do so. For example:   *Data cannot be shared publicly because of [XXX]. Data are available from the XXX Institutional Data Access / Ethics Committee (contact via XXX) for researchers who meet the criteria for access to confidential data.*  *The data underlying the results presented in the study are available from (include the name of the third party* | All data analyzed during this study are included in this article and can be accessed from the Office of the National Economic and Social Development Council (NESDC), Thailand, the United Nations University World Institute for Development Economics Research (UNU-WIDER), and the World Bank all of which are listed in References. |

*Powered by Editorial Manager® and ProduXion Manager® from Aries Systems Corporation*

| *and contact information or URL).*   - This text is appropriate if the data are owned by a third party and authors do not have permission to share the data.   * typeset |  |
| --- | --- |
| Additional data availability information: |  |

*Powered by Editorial Manager® and ProduXion Manager® from Aries Systems Corporation*

Manuscript	[Click here to access/download;Manuscript;Manuscript_PLOS ONE.docx](https://www.editorialmanager.com/pone/download.aspx?id=31740776&guid=b4eb7335-263a-4619-8d09-9e045c92be45&scheme=1)

# An investigation of the performance of parametric functional forms for the Lorenz curve

## Thitithep Sitthiyot^1^, Kanyarat Holasut^2^

^1^ Corresponding author, Department of Banking and Finance, Faculty of Commerce and Accountancy, Chulalongkorn University, Mahitaladhibesra Bld., 10^th^ Fl., Phayathai Rd., Pathumwan, Bangkok 10330, Thailand, E-mail address: [thitithep@cbs.chula.ac.th.,](mailto:thitithep@cbs.chula.ac.th) ORCID ID: https://orcid.org/0000-0001-9610-2279.

^2^ Department of Chemical Engineering, Faculty of Engineering, Khon Kaen University, Mittapap Rd., Muang District, Khon Kaen 40002, Thailand, E-mail address: [kanyarat@kku.ac.th.](mailto:kanyarat@kku.ac.th)

## Abstract

Given that an excellent performance of any parametric functional form for the Lorenz curve that is based on a single country case study and a limited range of distribution must be treated with great caution, this study investigates the performance of a single-parameter functional form proposed by Paul and Shankar (2020) who use income data of Australia to show that their functional form is superior to the other existing widely used functional forms considered in their study. By using both mathematical proof and empirical data of 40 countries around the world, this study demonstrates that Paul and Shankar (2020)’s functional form not only fails to fit the actual observations well but also is generally outperformed by the other popular functional forms considered in their study. Moreover, to overcome the limitation of the performance of a single-parameter functional form on the criterion of the estimated Gini index, this study employs a functional form that has more than one parameter in order to show that, by and large, it performs better than all popular single-parameter functional forms considered in

Paul and Shankar (2020)’s study. Thus, before applying any functional form to estimate the Lorenz curve, policymakers should check if it could describe the shape of income distributions of different countries through the changes in parameter values and yield the values of the estimated Gini index that are close to their observed data. Using a functional form that does not fit the actual observations could adversely affect inequality measures and income distribution policies.

## 1. Introduction

Paul and Shankar (2020) propose an alternative single-parameter functional form for the Lorenz curve. By utilizing the data on the Australian decile income shares between 2001 and 2010 that are constructed using the individual income data from the first 10 waves of Household, Income, and Labour Dynamics Australia (HILDA) Survey, they show that, on the basis of mean- squared error (MSE) and information inaccuracy measure (IIM), their functional form outperforms the other existing widely used functional forms, namely, Kakwani and Podder (1973), Aggarwal (1984), Chotikapanich (1993), and a functional form implied by Pareto distribution. Moreover, the Gini index in all 10 years, calculated using the functional form that they propose, rank second closely behind those estimated using Aggarwal (1984)’s functional form.

According to Jorda et al. (2021), an excellent performance of any parametric functional form for the Lorenz curve that is relied on a single country case study and a limited range of distribution should be treated with extreme caution. Jorda et al. (2021) also note that previous studies by Cowell and Mehta (1982) and Shorrocks and Wan (2008) also point out the similar caveat. Given the superiority of Paul and Shankar (2020)’s functional form over the other existing widely used functional forms, it is therefore worthwhile to conduct an examination to find out if we use grouped income data of other countries, the performance of Paul and Shankar (2020)’s functional form is *still* superior to the other existing widely used functional forms considered in their study. The findings from this investigation should also serve as a check-and- balance not only for economics but also for other scientific disciplines that use the Lorenz curve to analyze size distributions of non-negative quantities and inequality. This is because a good functional form for the Lorenz curve must describe the shape of income distributions of different

countries, regions, socioeconomic groups, and in different time periods through the changes in parameter values (Dagum, 1977). In addition, Dagum (1977) notes that it should provide a good fit of the entire range of income distribution since all observations are relevant for an accurate measurement of income inequality, supporting social and income policies, as well as determining taxation structure.

Our findings indicate that, despite its parsimony, the single-parameter functional form proposed by Paul and Shankar (2020) has two serious limitations. First, it fails to fit the actual observations well when the inequality in income distribution as measured by the Gini index is lower than the critical threshold which is found to be 0.4180. We verify the first limitation by demonstrating both mathematically and empirically that when a country’s Gini index is lower than 0.4180, not only does the functional form proposed by Paul and Shankar (2020) not perform well but also all other popular functional forms considered in their study, by and large, perform far better than Paul and Shankar (2020)’s specified functional form on the basis of the coefficient of determination (R^2^), MSE, IIM, and the estimated Gini index. The numerical example and the income data of 20 Organization for Economic Co-operation and Development (OECD) countries are employed in order to illustrate the first limitation. Second, on the basis of R^2^, MSE, IIM, and the estimated Gini index, the performance of Paul and Shankar (2020)’s functional specification, compared to the other existing widely used functional forms considered in their study, is at best mixed when the upper part of a country’s income distribution has a long-tailed property which is defined as the income share of the top 20% being greater than 50% of total income share. The income data of the other 20 different countries around the world whose upper part of income distribution has a long-tailed property are employed in order to illustrate the second limitation.

While Paul and Shankar (2020) show that the values of the Gini index estimated using the functional form that they propose rank second closely behind those estimated using Aggarwal (1984)’s functional form, this study would like to note that, to evaluate the performance of a functional form for the Lorenz curve on the basis of the estimated Gini index, a functional form that contains more than one parameter is required since the curvature of the estimated Lorenz curve has to be adjustable so that it would fit the actual data as much as possible while keeping the value of the estimated Gini index the same. According to Dagum (1977), this cannot be done by using a single-parameter functional form because the estimated Gini index would be a monotonic function of it. In order to illustrate that a functional form that has more than one parameter, by and large, gives the values of the estimated Gini index closer to the actual observations than single-parameter functional forms, a two-parameter functional form proposed by Sitthiyot and Holasut (2021) is employed in order to estimate the Lorenz curve and calculate the value of the estimated Gini index using the same set of grouped income data of 40 countries.

This study contains 4 sections. Following Introduction, Section 2 explains materials and methods used. Section 3 presents results and discussion. Finally, Section 4 concludes and provides some remarks when using any parametric functional form for the Lorenz curve to fit the actual income data.

## 2. Materials and methods

To examine the performance of Paul and Shankar (2020)’s functional form, let 𝑝 denote the cumulative normalized rank of income and (𝑝, 𝛾) denote the cumulative normalized income

for a given value of parameter 𝛾. The functional form for the Lorenz curve proposed by Paul and Shankar (2020) is as follows:

(𝑝, 𝛾) = 𝑝 ∗ [^𝑒^−𝛾(1−𝑒𝑝)−1] , 𝛾 > 0

−(1−𝑒)

𝑒	−1

(1)

Given that the value of parameter 𝛾 must be greater than 0, the first limitation of this functional form could be demonstrated mathematically by setting the value of parameter 𝛾 to be 0 and then using equation (1) to calculate the values of 𝐿(𝑝, 𝛾) for different values of 𝑝 which are 0.0, 0.1, 0.2, 0.3, 0.4, 0.5, 0.6, 0.7, 0.8, 0.9, and 1.0. All estimated values of (𝑝, 𝛾) would be used to calculate the area under the Lorenz curve using the numerical integration since the above specified functional form does not have a closed-form expression for the Gini index. The next step is to compute the value of the Gini index that corresponds to the value of parameter 𝛾 = 0.

The value of the Gini index, given 𝛾 = 0, would be the critical threshold where the specified functional form would not be able to fit the actual observations well below this critical threshold since the value of parameter 𝛾 cannot be 0 or negative.

To illustrate the first limitation of Paul and Shankar (2020)’s functional form empirically as well as to evaluate its performance compared to those of the other popular functional specifications considered in their study, namely, Kakwani and Podder (1973), Aggarwal (1984), Chotikapanich (1993), and a functional form implied by Pareto distribution, this study uses the data on the observed Gini index and the decile income shares of 20 OECD countries from the United Nations University-World Income Inequality Database (UNU-WIID). The reason that we choose the OECD countries is because the values of the observed Gini index of these countries are lower than the critical threshold. We would like to note that, based on the mathematical proof as described above, this critical threshold should apply to not only income distribution of any

country and in any time period but also income distribution generated by using any simulation method as long as the value of the Gini index is less than 0.4180, not just to the income distributions of 20 OECD countries employed in this study.

For the second limitation of Paul and Shankar (2020)’s functional form where it does not perform well compared to the other popular functional forms when a country’s income distribution has a long-tailed property, the data on the observed Gini index and the income shares by decile of the other 20 different countries around the world from the Office of the National Economic and Social Development Council (NESDC), Thailand, the UNU-WIID, and the World Bank are used to illustrate this limitation. These countries are chosen because the upper part of income distribution is long-tailed where the top 20% of income earners receive more than 50% of the total income as defined in Introduction.

In this study, the performance of functional form proposed by Paul and Shankar (2020) is compared to the other existing widely used functional specifications considered in their study which are Kakwani and Podder (1973), Aggarwal (1984), Chotikapanich (1993), and a functional form implied by Pareto distribution. These functional forms are expressed as follows:

Kakwani and Podder (1973): (𝑝, 𝛿) = 𝑝𝑒^−𝛿(1−𝑝)^, 𝛿 > 0	(2)

Aggarwal (1984): (𝑝, 𝜃) =	^(1−𝜃)^2𝑝

(	)^2^

1+𝜃	−4𝜃𝑝

, 0 < 𝜃 < 1	(3)

Chotikapanich (1993): (𝑝, 𝑘) = ^𝑒^𝑘𝑝−1 , 𝑘 > 0	(4)

𝑘

−1

1

Functional form implied by Pareto distribution: (𝑝, 𝛼) = 1 − (1 − 𝑝)𝛼, 𝛼 > 1	(5)

Paul and Shankar (2020, p. 1396) note that their alternative functional form for the Lorenz curve, Kakwani and Podder (1973)’s functional form, and a functional form implied by Pareto distribution do not have a closed-form expression for the Gini index, hence the estimated

Gini index has to be calculated by using the numerical integration. Provided that a good functional form for the Lorenz curve should have a closed-form expression for the Gini index as noted by Dagum (1977), this study finds out that only does Paul and Shankar (2020)’s functional form not have an explicit mathematical solution for the Gini index. For the other existing widely used functional forms considered in their study, the Gini index can be directly computed since these functional forms have closed-form expressions for the Gini index which are as follows:

Kakwani and Podder (1973): Gini index = 1 − ^2(𝛿−1)^ − ^2𝑒^−𝛿

(6)

𝛿2	𝛿2

Aggarwal (1984): Gini index = (1+𝜃)2 [^(1−𝜃)2^ 𝑙𝑛 (^1−𝜃^ 2

(7)

2𝜃

4𝜃

1+𝜃)

+ 1] − 1

Chotikapanich (1993): Gini index = ^(𝑘−2)^𝑘+(𝑘+2)

𝑘

(𝑒 −1)

Functional form implied by Pareto distribution: Gini index = ^𝛼−1^

𝛼+1

(8)

(9)

This study uses the curve fitting technique based on minimizing error sum of squares to estimate the Lorenz curve. The statistical measures of goodness-of-fit, namely, MSE and IIM are employed for assessing the performance of all functional forms, both of which are the same criteria as used in Paul and Shankar (2020)’s study. In addition, this study uses R^2^ and the estimated Gini index to evaluate the performance of all functional forms. The closer the value of R^2^ is to 1 and the closer the value of MSE is to zero, the better the estimated functional form. For the IIM criterion, the estimated functional specification that has a smaller absolute value of IIM is better than those with larger absolute values of IIM. In this study, the Microsoft Excel Data Analysis program and the Microsoft Excel Solver program are used for calculating the descriptive statistics on the Gini index and the income shares by decile as well as estimating the parameters and calculating the estimated Gini index. As suggested by Dagum (1977), from a

perspective of computational cost and the acceptance of the specified functional form in practice, a simple method of parameter estimation is always an advantage. Table 1 reports the descriptive statistics of the observed Gini index and the decile income shares of 20 OECD countries whereas those of the other 20 countries are reported in Table 2. The next section presents our results and provides discussion.

## Table 1. The descriptive statistics of the observed Gini index and the decile income shares of 20 OECD countries whose values of the observed Gini index are below the critical threshold of 0.4180.

**Indicator	Observed**

|  | **Gini index** | **D1** | **D2** | **D3** | **D4** | **D5** | **D6** | **D7** | **D8** | **D9** | **D10** |
| --- | --- | --- | --- | --- | --- | --- | --- | --- | --- | --- | --- |
| **Mean** | 0.2810 | 0.0326 | 0.0536 | 0.0646 | 0.0744 | 0.0843 | 0.0948 | 0.1065 | 0.1212 | 0.1434 | 0.2247 |
| **Median** | 0.2805 | 0.0320 | 0.0540 | 0.0645 | 0.0745 | 0.0840 | 0.0950 | 0.1065 | 0.1210 | 0.1420 | 0.2255 |
| **Mode** | 0.3090 | 0.0320 | 0.0580 | 0.0640 | 0.0760 | 0.0850 | 0.0950 | 0.1080 | 0.1210 | 0.1420 | 0.2240 |
| **Minimum** | 0.2320 | 0.0200 | 0.0460 | 0.0570 | 0.0680 | 0.0790 | 0.0910 | 0.1020 | 0.1160 | 0.1360 | 0.1910 |
| **Maximum** | 0.3270 | 0.6510 | 0.0620 | 0.0740 | 0.0820 | 0.0930 | 0.1000 | 0.1110 | 0.1300 | 0.1590 | 0.2450 |
| **Standard deviation** | 0.0268 | 0.0053 | 0.0046 | 0.0044 | 0.0037 | 0.0032 | 0.0024 | 0.0024 | 0.0033 | 0.0061 | 0.0152 |
| **Number of countries** | 20 | 20 | 20 | 20 | 20 | 20 | 20 | 20 | 20 | 20 | 20 |

**Decile income shares**

## Table 2. The descriptive statistics of the observed Gini index and the decile income shares of the other 20 countries whose upper part of the income distribution has a long-tailed property defined as the income share of the top 20% being greater than 50% of total income share.

**Indicator	Income share**

|  | **of the top 20%** | **Gini index** | **D1** | **D2** | **D3** | **D4** | **D5** | **D6** | **D7** | **D8** | **D9** | **D10** |
| --- | --- | --- | --- | --- | --- | --- | --- | --- | --- | --- | --- | --- |
| **Mean** | 0.5576 | 0.5065 | 0.0140 | 0.0263 | 0.0363 | 0.0455 | 0.0561 | 0.0688 | 0.0852 | 0.1100 | 0.1545 | 0.4032 |
| **Median** | 0.5541 | 0.5070 | 0.0126 | 0.0257 | 0.0348 | 0.0452 | 0.0561 | 0.0703 | 0.0858 | 0.1110 | 0.1572 | 0.3989 |
| **Mode** | N/A | 0.5400 | 0.0100 | 0.0260 | 0.0342 | N/A | 0.0600 | 0.0680 | 0.0870 | 0.1150 | 0.1510 | N/A |
| **Minimum** | 0.5112 | 0.4470 | 0.0090 | 0.0179 | 0.0250 | 0.0331 | 0.0428 | 0.0556 | 0.0742 | 0.0979 | 0.1331 | 0.3529 |
| **Maximum** | 0.6374 | 0.5907 | 0.0264 | 0.0375 | 0.0452 | 0.0537 | 0.0646 | 0.0769 | 0.0933 | 0.1180 | 0.1690 | 0.4725 |
| **Standard deviation** | 0.0322 | 0.0359 | 0.0043 | 0.0043 | 0.0047 | 0.0049 | 0.0050 | 0.0051 | 0.0053 | 0.0060 | 0.0108 | 0.0328 |
| **Number of countries** | 20 | 20 | 20 | 20 | 20 | 20 | 20 | 20 | 20 | 20 | 20 | 20 |

**Observed**

**Decile income shares**

## 3. Results and discussion

### 3.1 The mathematical proof of the critical threshold for the Gini index

This study first demonstrates the mathematical proof of the first limitation of Paul and Shankar (2020)’s functional form. According to their specified functional form with the values of

𝑝 being equal to 0.0, 0.1, 0.2, 0.3, 0.4, 0.5, 0.6, 0.7, 0.8, 0.9, and 1.0, Table 3 reports different values of the Gini index (rounding up to 7 decimal places) for different values of parameter 𝛾 ranging between −0.000001 and 2.0.

## Table 3. The values of the Gini index calculated using different values of parameter

𝜸 **based on Paul and Shankar (2020)’s functional form.**

| **Parameter** 𝜸 | **Gini index** |
| --- | --- |
| -0.000001 | 0.4179801 |
| **0** | **0.4179803** |
| 0.000001 | 0.4179804 |
| 0.00001 | 0.4179818 |
| 0.0001 | 0.4179955 |
| 0.001 | 0.4181326 |
| 0.01 | 0.4195044 |
| 0.1 | 0.4332468 |
| 0.5 | 0.4941095 |
| 1.0 | 0.5665420 |
| 1.25 | 0.6000009 |
| 1.5 | 0.6311545 |

2.0	0.6859776

The numerical results in Table 3 indicate that when the value of parameter 𝛾 is equal to 0 (the average value of parameter 𝛾 when 𝛾 = −0.000001 and 0.000001), the critical threshold for the Gini index is equal to 0.4179803 (the average value of the Gini index when the Gini index = 0.4179801 and 0.4179804). Let the value of the critical threshold for the Gini index = 0.4180 for the purpose of demonstration. This implies that when the level of inequality in income distribution as measured by the observed Gini index is less than the critical threshold, using Paul

and Shankar (2020)’s functional form for approximating the Lorenz curve and calculating the Gini index would result in the value of the estimated Gini index being equal to 0.4180. Provided that this is the mathematical proof, it is thus always true not only for income distribution of any country and in any time period but also income distribution generated by using any simulation technique so long as the value of the Gini index is less than 0.4180.

### 3.2 The performance comparison between Paul and Shankar (2020)’s functional form and the other popular functional forms when the Gini index is below the critical threshold

In order to show the first limitation of Paul and Shankar (2020)’s functional form empirically, this study employs the data on decile income shares of 20 OECD countries from the UNU-WIID and estimate the Lorenz curve using their functional form and the other existing widely used functional specifications considered in their study, namely, Kakwani and Podder (1973), Aggarwal (1984), Chotikapanich (1993), and a functional form implied by Pareto distribution. The estimated values of parameters for each functional form are reported in Table A1 in Appendix. We then compare the performance of Paul and Shankar (2020)’s functional form to those of the other existing widely used functional forms on the criteria of R^2^, MSE, IIM, and the estimated Gini index. Table 4a – 4d reports our results.

**Table 4a. The income data of 20 OECD countries are used to demonstrate the performance of Paul and Shankar (2020)’s functional form and those of the other popular functional forms based on R^2^.** The bold numbers (if any) indicate that Paul and Shankar (2020)’s functional form is superior to the other existing widely used functional forms.

|  | | | **PS**^a^ **KP**^b^ **A**^c^ | | | **C**^d^ **Pareto**^e^ | |
| --- | --- | --- | --- | --- | --- | --- | --- |
| Slovakia | 2017 | 0.2320 | 0.8760 | 0.9989 | 0.9986 | 0.9988 | 0.9918 |
| Slovenia | 2017 | 0.2370 | 0.8827 | 0.9990 | 0.9993 | 0.9992 | 0.9931 |
| Czech Republic | 2017 | 0.2450 | 0.8851 | 0.9974 | 0.9989 | 0.9978 | 0.9958 |
| Finland | 2017 | 0.2530 | 0.8958 | 0.9975 | 0.9990 | 0.9980 | 0.9951 |
| Belgium | 2017 | 0.2600 | 0.9094 | 0.9989 | 0.9992 | 0.9991 | 0.9915 |
| Norway | 2017 | 0.2610 | 0.9039 | 0.9975 | 0.9984 | 0.9978 | 0.9934 |
| Netherlands | 2017 | 0.2710 | 0.9151 | 0.9978 | 0.9989 | 0.9982 | 0.9933 |
| Denmark | 2017 | 0.2760 | 0.9151 | 0.9967 | 0.9983 | 0.9972 | 0.9940 |
| Austria | 2017 | 0.2790 | 0.9244 | 0.9978 | 0.9987 | 0.9981 | 0.9921 |
| Sweden | 2017 | 0.2800 | 0.9245 | 0.9979 | 0.9987 | 0.9982 | 0.9918 |
| Hungary | 2017 | 0.2810 | 0.9270 | 0.9979 | 0.9989 | 0.9983 | 0.9920 |
| Germany | 2017 | 0.2910 | 0.9346 | 0.9974 | 0.9989 | 0.9979 | 0.9921 |
| Poland | 2017 | 0.2920 | 0.9349 | 0.9971 | 0.9987 | 0.9977 | 0.9924 |
| France | 2017 | 0.2930 | 0.9295 | 0.9950 | 0.9978 | 0.9958 | 0.9948 |
| Switzerland | 2017 | 0.3010 | 0.9394 | 0.9959 | 0.9983 | 0.9966 | 0.9934 |
| Ireland | 2017 | 0.3060 | 0.9464 | 0.9966 | 0.9990 | 0.9974 | 0.9926 |
| Canada | 2017 | 0.3090 | 0.9532 | 0.9983 | 0.9988 | 0.9986 | 0.9885 |
| Luxembourg | 2017 | 0.3090 | 0.9524 | 0.9978 | 0.9991 | 0.9983 | 0.9899 |
| Estonia | 2017 | 0.3160 | 0.9618 | 0.9991 | 0.9989 | 0.9993 | 0.9857 |
| Italy | 2017 | 0.3270 | 0.9639 | 0.9978 | 0.9982 | 0.9981 | 0.9869 |

**Country	Year	Observed Gini index	Coefficient of determination (R^2^)**

Note: ^a^ PS = Paul and Shankar (2020), ^b^ KP = Kakwani and Podder (1973), ^c^ A = Aggarwal (1984), ^d^ C= Chotikapanich (1993), ^e^ Pareto = Functional form implied by Pareto distribution.

**Table 4b. The income data of 20 OECD countries are used to demonstrate the performance of Paul and Shankar (2020)’s functional form and those of the other popular functional forms based on MSE.** The bold numbers (if any) indicate that Paul and Shankar (2020)’s functional form is superior to the other existing widely used functional forms.

| **Country** | **Year Observed Gini index Mean-squared error (MSE)**  **PS**^a^ **KP**^b^ **A**^c^ **C**^d^ **Pareto**^e^ | | | | | | |
| --- | --- | --- | --- | --- | --- | --- | --- |
| Slovakia | 2017 | 0.2320 | 0.0012 | 0.0001 | 0.0001 | 0.0001 | 0.0003 |
| Slovenia | 2017 | 0.2370 | 0.0011 | 0.0001 | 0.0001 | 0.0001 | 0.0003 |
| Czech Republic | 2017 | 0.2450 | 0.0011 | 0.0002 | 0.0001 | 0.0002 | 0.0002 |
| Finland | 2017 | 0.2530 | 0.0010 | 0.0002 | 0.0001 | 0.0002 | 0.0002 |
| Belgium | 2017 | 0.2600 | 0.0009 | 0.0001 | 0.0001 | 0.0001 | 0.0004 |
| Norway | 2017 | 0.2610 | 0.0010 | 0.0002 | 0.0002 | 0.0002 | 0.0003 |
| Netherlands | 2017 | 0.2710 | 0.0008 | 0.0002 | 0.0001 | 0.0002 | 0.0003 |
| Denmark | 2017 | 0.2760 | 0.0009 | 0.0003 | 0.0002 | 0.0003 | 0.0002 |
| Austria | 2017 | 0.2790 | 0.0008 | 0.0002 | 0.0001 | 0.0002 | 0.0003 |
| Sweden | 2017 | 0.2800 | 0.0008 | 0.0002 | 0.0001 | 0.0002 | 0.0003 |
| Hungary | 2017 | 0.2810 | 0.0008 | 0.0002 | 0.0001 | 0.0002 | 0.0004 |
| Germany | 2017 | 0.2910 | 0.0007 | 0.0002 | 0.0001 | 0.0002 | 0.0003 |
| Poland | 2017 | 0.2920 | 0.0007 | 0.0002 | 0.0001 | 0.0002 | 0.0003 |
| France | 2017 | 0.2930 | 0.0009 | 0.0004 | 0.0002 | 0.0004 | 0.0002 |
| Switzerland | 2017 | 0.3010 | 0.0008 | 0.0004 | 0.0002 | 0.0003 | 0.0003 |
| Ireland | 2017 | 0.3060 | 0.0006 | 0.0003 | 0.0001 | 0.0002 | 0.0004 |
| Canada | 2017 | 0.3090 | 0.0005 | 0.0002 | 0.0001 | 0.0002 | 0.0005 |
| Luxembourg | 2017 | 0.3090 | 0.0006 | 0.0002 | 0.0001 | 0.0002 | 0.0005 |
| Estonia | 2017 | 0.3160 | 0.0004 | 0.0001 | 0.0001 | 0.0001 | **0.0007** |
| Italy | 2017 | 0.3270 | 0.0005 | 0.0002 | 0.0001 | 0.0002 | **0.0006** |

Note: ^a^ PS = Paul and Shankar (2020), ^b^ KP = Kakwani and Podder (1973), ^c^ A = Aggarwal (1984), ^d^ C= Chotikapanich (1993), ^e^ Pareto = Functional form implied by Pareto distribution.

**Table 4c. The income data of 20 OECD countries are used to demonstrate the performance of Paul and Shankar (2020)’s functional form and those of the other popular functional forms based on IIM.** The bold numbers (if any) indicate that Paul and Shankar (2020)’s functional form is superior to the other existing widely used functional forms.

| **Country** | **Year Observed Gini index Information inaccuracy measure (IIM)**  **PS**^a^ **KP**^b^ **A**^c^ **C**^d^ **Pareto**^e^ | | | | | | |
| --- | --- | --- | --- | --- | --- | --- | --- |
| Slovakia | 2017 | 0.2320 | 0.1014 | 0.0077 | 0.0087 | 0.0079 | 0.0201 |
| Slovenia | 2017 | 0.2370 | 0.1045 | 0.0046 | 0.0044 | 0.0045 | 0.0148 |
| Czech Republic | 2017 | 0.2450 | 0.1078 | 0.0050 | 0.0025 | 0.0043 | 0.0062 |
| Finland | 2017 | 0.2530 | 0.1016 | 0.0056 | 0.0032 | 0.0049 | 0.0083 |
| Belgium | 2017 | 0.2600 | 0.0850 | 0.0041 | 0.0040 | 0.0040 | 0.0168 |
| Norway | 2017 | 0.2610 | 0.0885 | 0.0095 | 0.0084 | 0.0092 | 0.0150 |
| Netherlands | 2017 | 0.2710 | 0.0845 | 0.0070 | 0.0052 | 0.0065 | 0.0139 |
| Denmark | 2017 | 0.2760 | 0.0854 | 0.0106 | 0.0082 | 0.0100 | 0.0131 |
| Austria | 2017 | 0.2790 | 0.0762 | 0.0089 | 0.0077 | 0.0086 | 0.0180 |
| Sweden | 2017 | 0.2800 | 0.0753 | 0.0076 | 0.0065 | 0.0073 | 0.0168 |
| Hungary | 2017 | 0.2810 | 0.0751 | 0.0084 | 0.0070 | 0.0080 | 0.0182 |
| Germany | 2017 | 0.2910 | 0.0710 | 0.0077 | 0.0056 | 0.0071 | 0.0162 |
| Poland | 2017 | 0.2920 | 0.0704 | 0.0085 | 0.0061 | 0.0078 | 0.0164 |
| France | 2017 | 0.2930 | 0.0825 | 0.0142 | 0.0095 | 0.0130 | 0.0119 |
| Switzerland | 2017 | 0.3010 | 0.0707 | 0.0102 | 0.0062 | 0.0091 | 0.0127 |
| Ireland | 2017 | 0.3060 | 0.0693 | 0.0093 | 0.0052 | 0.0081 | 0.0158 |
| Canada | 2017 | 0.3090 | 0.0518 | 0.0053 | 0.0049 | 0.0050 | 0.0229 |
| Luxembourg | 2017 | 0.3090 | 0.0546 | 0.0046 | 0.0029 | 0.0040 | 0.0188 |
| Estonia | 2017 | 0.3160 | 0.0429 | 0.0029 | 0.0040 | 0.0029 | 0.0297 |
| Italy | 2017 | 0.3270 | 0.0423 | 0.0099 | 0.0102 | 0.0098 | 0.0296 |

Note: ^a^ PS = Paul and Shankar (2020), ^b^ KP = Kakwani and Podder (1973), ^c^ A = Aggarwal (1984), ^d^ C= Chotikapanich (1993), ^e^ Pareto = Functional form implied by Pareto distribution.

**Table 4d. The income data of 20 OECD countries are used to demonstrate the performance of Paul and Shankar (2020)’s functional form and those of the other popular functional forms based on the estimated Gini index.** The bold numbers (if any) indicate that Paul and Shankar (2020)’s functional form is superior to the other existing widely used functional forms.

| **Country** | **Year Observed Gini index Estimated Gini index**  **PS**^a^ **KP**^b^ **A**^c^ **C**^d^ **Pareto**^e^ | | | | | | |
| --- | --- | --- | --- | --- | --- | --- | --- |
| Slovakia | 2017 | 0.2320 | 0.4180 | 0.2242 | 0.2227 | 0.2239 | 0.2171 |
| Slovenia | 2017 | 0.2370 | 0.4180 | 0.2315 | 0.2305 | 0.2313 | 0.2250 |
| Czech Republic | 2017 | 0.2450 | 0.4180 | 0.2386 | 0.2389 | 0.2387 | 0.2351 |
| Finland | 2017 | 0.2530 | 0.4180 | 0.2470 | 0.2470 | 0.2470 | 0.2426 |
| Belgium | 2017 | 0.2600 | 0.4180 | 0.2540 | 0.2526 | 0.2538 | 0.2463 |
| Norway | 2017 | 0.2610 | 0.4180 | 0.2520 | 0.2514 | 0.2518 | 0.2467 |
| Netherlands | 2017 | 0.2710 | 0.4180 | 0.2628 | 0.2622 | 0.2627 | 0.2568 |
| Denmark | 2017 | 0.2760 | 0.4180 | 0.2646 | 0.2644 | 0.2646 | 0.2599 |
| Austria | 2017 | 0.2790 | 0.4180 | 0.2711 | 0.2701 | 0.2709 | 0.2644 |
| Sweden | 2017 | 0.2800 | 0.4180 | 0.2709 | 0.2697 | 0.2706 | 0.2639 |
| Hungary | 2017 | 0.2810 | 0.4180 | 0.2739 | 0.2728 | 0.2737 | 0.2669 |
| Germany | 2017 | 0.2910 | 0.4180 | 0.2833 | 0.2824 | 0.2831 | 0.2764 |
| Poland | 2017 | 0.2920 | 0.4180 | 0.2843 | 0.2835 | 0.2842 | 0.2777 |
| France | 2017 | 0.2930 | 0.4180 | 0.2834 | 0.2837 | 0.2834 | 0.2794 |
| Switzerland | 2017 | 0.3010 | 0.4180 | 0.2927 | 0.2924 | 0.2926 | 0.2871 |
| Ireland | 2017 | 0.3060 | 0.4180 | 0.3002 | 0.2995 | 0.3001 | 0.2933 |
| Canada | 2017 | 0.3090 | 0.4180 | 0.3027 | 0.3004 | 0.3022 | 0.2929 |
| Luxembourg | 2017 | 0.3090 | 0.4180 | 0.3037 | 0.3019 | 0.3034 | 0.2948 |
| Estonia | 2017 | 0.3160 | 0.4180 | 0.3120 | 0.3087 | 0.3114 | 0.2999 |
| Italy | 2017 | 0.3270 | 0.4180 | 0.3183 | 0.3156 | 0.3178 | 0.3079 |

Note: ^a^ PS = Paul and Shankar (2020), ^b^ KP = Kakwani and Podder (1973), ^c^ A = Aggarwal (1984), ^d^ C= Chotikapanich (1993), ^e^ Pareto = Functional form implied by Pareto distribution.

The overall results in Table 4a – 4c suggest that, on the criteria of R^2^, MSE, and IIM, all other popular functional forms perform far better than the functional form proposed by Paul and Shankar (2020). With regard to the value of the estimated Gini index, all results in Table 4d clearly confirm the first limitation of Paul and Shankar (2020)’s functional form in that when the observed Gini index is lower than the critical threshold, using Paul and Shankar (2020)’s functional form to estimate the Lorenz curve and compute the Gini index would result in the estimated Gini index being equal to 0.4180. Provided that the parameter 𝛾 cannot take value below 0, this study sets the value of estimated parameter 𝛾 to be equal to 0.0000001 which would result in the estimated value of the Gini index to be 0.4180 for all 20 OECD countries.

The results in Table 4d also show that the values of the estimated Gini index calculated using the other popular functional forms, namely, Kakwani and Podder (1973), Aggarwal (1984), Chotikapanich (1993), and a functional form implied by Pareto distribution are much closer to the actual observations than those calculated using Paul and Shankar (2020)’s functional specification. The performance comparison between Paul and Shankar (2020)’s functional forms and the other existing widely used functional forms considered in their study when the observed Gini index is less than the critical threshold of 0.4180 is also illustrated in Fig. 1.

## Fig. 1. The performance comparison between Paul and Shankar (2020)’s functional form and the other existing widely used functional forms, namely, Kakwani and Podder (1973), Aggarwal (1984), Chotikapanich (1993), and functional form implied by Pareto distribution when the observed Gini index is less than the critical threshold of 0.4180. (a) R^2^. (b) MSE. (c) IIM. (d) Estimated Gini index.

### 3.3 The performance comparison between Paul and Shankar (2020)’s functional form and the other popular functional forms when the upper part of income distribution has a long-tailed property

To demonstrate the second limitation of Paul and Shankar (2020)’s functional specification, this study employs the data on income shares by decile of the other 20 different countries around the world from the NESDC, the UNU-WIID, and the World Bank to estimate the Lorenz curve. Table A2 in Appendix reports the estimated values of parameters for each functional form. We then evaluate the performance of Paul and Shankar (2020)’s functional form

as well as those of the other existing widely used functional forms considered in their study based on R^2^, MSE, IIM, and the estimated Gini index. Our results are reported in Table 5a – 5d.

**Table 5a. The income data of the other 20 countries are used to demonstrate the performance of Paul and Shankar (2020)’s functional form and those of the other popular functional forms based on R^2^.** The bold numbers (if any) indicate that Paul and Shankar (2020)’s functional form is superior to the other existing widely used functional forms.

**Country	Year	Income share**

**Coefficient of determination (R^2^)**

Note: ^a^ PS = Paul and Shankar (2020), ^b^ KP = Kakwani and Podder (1973), ^c^ A = Aggarwal (1984), ^d^ C= Chotikapanich (1993), ^e^ Pareto = Functional form implied by Pareto distribution.

| **of the top 20%** | | | **PS**^a^ **KP**^b^ **A**^c^ | | | **C**^d^ **Pareto**^e^ | |
| --- | --- | --- | --- | --- | --- | --- | --- |
| Thailand | 2017 | 51.12 | 0.9805 | 0.9875 | 0.9978 | 0.9899 | 0.9888 |
| Malawi | 2017 | 51.67 | 0.9608 | 0.9724 | 0.9911 | 0.9762 | 0.9969 |
| Ecuador | 2015 | 51.90 | 0.9801 | 0.9866 | 0.9977 | 0.9891 | 0.9888 |
| Benin | 2015 | 52.13 | 0.9811 | 0.9858 | 0.9958 | 0.9880 | 0.9862 |
| Philippines | 2012 | 52.68 | 0.9788 | 0.9855 | 0.9980 | 0.9883 | 0.9896 |
| Sri Lanka | 2010 | 54.40 | 0.9743 | 0.9795 | 0.9953 | 0.9826 | 0.9909 |
| Nicaragua | 2014 | 54.50 | 0.9772 | 0.9817 | 0.9962 | 0.9847 | 0.9893 |
| Bangladesh | 2016 | 54.75 | 0.9682 | 0.9736 | 0.9926 | 0.9772 | 0.9928 |
| Costa Rica | 2016 | 54.90 | 0.9842 | 0.9878 | 0.9986 | 0.9902 | 0.9847 |
| Saint Lucia | 2016 | 55.39 | 0.9857 | 0.9883 | 0.9982 | 0.9906 | **0.9827** |
| Paraguay | 2016 | 55.41 | 0.9824 | 0.9855 | 0.9978 | 0.9882 | 0.9857 |
| Mexico | 2016 | 55.70 | 0.9738 | 0.9783 | 0.9958 | 0.9817 | 0.9912 |
| India | 2012 | 56.38 | 0.9810 | 0.9839 | 0.9979 | 0.9868 | 0.9866 |
| Eswatini | 2010 | 56.73 | 0.9813 | 0.9842 | 0.9984 | 0.9871 | 0.9865 |
| Colombia | 2015 | 56.80 | 0.9815 | 0.9838 | 0.9977 | 0.9866 | 0.9859 |
| Brazil | 2018 | 58.60 | 0.9784 | 0.9797 | 0.9969 | 0.9829 | 0.9876 |
| Egypt | 2013 | 58.78 | 0.9726 | 0.9742 | 0.9953 | 0.9779 | 0.9908 |
| Mozambique | 2015 | 59.53 | 0.9647 | 0.9664 | 0.9929 | 0.9708 | 0.9943 |
| Honduras | 2012 | 60.10 | 0.9728 | 0.9734 | 0.9957 | 0.9772 | 0.9908 |
| Namibia | 2016 | 63.74 | 0.9810 | **0.9790** | 0.9985 | 0.9822 | 0.9854 |

**Table 5b. The income data of the other 20 countries are used to demonstrate the performance of Paul and Shankar (2020)’s functional form and those of the other popular functional forms based on MSE.** The bold numbers (if any) indicate that Paul and Shankar (2020)’s functional form is superior to the other existing widely used functional forms.

**Country	Year	Income share**

**of the top 20%**

**Mean-squared error (MSE)	PS**^a^	**KP**^b^	**A**^c^	**C**^d^	**Pareto**^e^

| Thailand | 2017 | 51.12 | 0.0013 | 0.0011 | 0.0003 | 0.0009 | 0.0006 |
| --- | --- | --- | --- | --- | --- | --- | --- |
| Malawi | 2017 | 51.67 | 0.0024 | 0.0021 | 0.0009 | 0.0019 | 0.0001 |
| Ecuador | 2015 | 51.90 | 0.0013 | 0.0011 | 0.0003 | 0.0010 | 0.0006 |
| Benin | 2015 | 52.13 | 0.0016 | 0.0015 | 0.0005 | 0.0013 | 0.0005 |
| Philippines | 2012 | 52.68 | 0.0013 | 0.0011 | 0.0002 | 0.0010 | 0.0006 |
| Sri Lanka | 2010 | 54.40 | 0.0019 | 0.0018 | 0.0006 | 0.0017 | 0.0004 |
| Nicaragua | 2014 | 54.50 | 0.0017 | 0.0017 | 0.0005 | 0.0015 | 0.0005 |
| Bangladesh | 2016 | 54.75 | 0.0025 | 0.0024 | 0.0009 | 0.0022 | 0.0003 |
| Costa Rica | 2016 | 54.90 | 0.0011 | 0.0010 | 0.0002 | 0.0009 | 0.0009 |
| Saint Lucia | 2016 | 55.39 | 0.0011 | 0.0011 | 0.0002 | 0.0009 | 0.0010 |
| Paraguay | 2016 | 55.41 | 0.0013 | 0.0013 | 0.0003 | 0.0012 | 0.0008 |
| Mexico | 2016 | 55.70 | 0.0019 | 0.0018 | 0.0005 | 0.0016 | 0.0004 |
| India | 2012 | 56.38 | 0.00137 | **0.00138** | 0.0003 | 0.0012 | 0.0008 |
| Eswatini | 2010 | 56.73 | 0.00127 | **0.00129** | 0.0002 | 0.0011 | 0.0008 |
| Colombia | 2015 | 56.80 | 0.00145 | **0.00149** | 0.0003 | 0.0013 | 0.0008 |
| Brazil | 2018 | 58.60 | 0.0017 | **0.0018** | 0.0004 | 0.0017 | 0.0006 |
| Egypt | 2013 | 58.78 | 0.0021 | **0.0023** | 0.0006 | 0.0021 | 0.0004 |
| Mozambique | 2015 | 59.53 | 0.0026 | **0.0028** | 0.0008 | 0.0025 | 0.0003 |
| Honduras | 2012 | 60.10 | 0.0021 | **0.0023** | 0.0005 | 0.0021 | 0.0005 |
| Namibia | 2016 | 63.74 | 0.0015 | **0.0018** | 0.0002 | **0.0016** | 0.0010 |

Note: ^a^ PS = Paul and Shankar (2020), ^b^ KP = Kakwani and Podder (1973), ^c^ A = Aggarwal (1984), ^d^ C= Chotikapanich (1993), ^e^ Pareto = Functional form implied by Pareto distribution.

**Table 5c. The income data of the other 20 countries are used to demonstrate the performance of Paul and Shankar (2020)’s functional form and those of the other popular functional forms based on IIM.** The bold numbers (if any) indicate that Paul and Shankar (2020)’s functional form is superior to the other existing widely used functional forms.

**Country	Year	Income share**

| **of the top 20% PS**a **KP**b **A**c | | | | | | **C**^d^ **Pareto**^e^ | |
| --- | --- | --- | --- | --- | --- | --- | --- |
| Thailand | 2017 | 51.12 | 0.0494 | 0.0280 | 0.0085 | 0.0235 | 0.0237 |
| Malawi | 2017 | 51.67 | 0.0930 | 0.0577 | 0.0226 | 0.0505 | 0.0064 |
| Ecuador | 2015 | 51.90 | 0.0475 | 0.0278 | 0.0068 | 0.0230 | 0.0225 |
| Benin | 2015 | 52.13 | 0.0464 | 0.0377 | 0.0210 | 0.0346 | 0.0338 |
| Philippines | 2012 | 52.68 | 0.0556 | 0.0312 | 0.0064 | 0.0254 | 0.0223 |
| Sri Lanka | 2010 | 54.40 | 0.0631 | 0.0466 | 0.0162 | 0.0406 | 0.0202 |
| Nicaragua | 2014 | 54.50 | 0.0545 | 0.0411 | 0.0139 | 0.0357 | 0.0237 |
| Bangladesh | 2016 | 54.75 | 0.0750 | 0.0617 | 0.0266 | 0.0554 | 0.0190 |
| Costa Rica | 2016 | 54.90 | 0.0416 | 0.0291 | 0.0079 | 0.0241 | 0.0360 |
| Saint Lucia | 2016 | 55.39 | 0.0352 | 0.0284 | 0.0107 | 0.0244 | **0.0418** |
| Paraguay | 2016 | 55.41 | 0.0448 | 0.0340 | 0.0101 | 0.0288 | 0.0325 |
| Mexico | 2016 | 55.70 | 0.0648 | 0.0482 | 0.0134 | 0.0411 | 0.0195 |
| India | 2012 | 56.38 | 0.0475 | 0.0368 | 0.0092 | 0.0309 | 0.0312 |
| Eswatini | 2010 | 56.73 | 0.0491 | 0.0356 | 0.0061 | 0.0290 | 0.0304 |
| Colombia | 2015 | 56.80 | 0.0458 | 0.0372 | 0.0097 | 0.0314 | 0.0317 |
| Brazil | 2018 | 58.60 | 0.0551 | 0.0486 | 0.0141 | 0.0419 | 0.0309 |
| Egypt | 2013 | 58.78 | 0.0675 | 0.0593 | 0.0165 | 0.0514 | 0.0220 |
| Mozambique | 2015 | 59.53 | 0.0871 | 0.0747 | 0.0195 | 0.0645 | 0.0130 |
| Honduras | 2012 | 60.10 | 0.0697 | 0.0623 | 0.0153 | 0.0535 | 0.0231 |
| Namibia | 2016 | 63.74 | 0.0522 | **0.0524** | 0.0063 | 0.0432 | 0.0365 |

**Information inaccuracy measure (IIM)**

Note: ^a^ PS = Paul and Shankar (2020), ^b^ KP = Kakwani and Podder (1973), ^c^ A = Aggarwal (1984), ^d^ C= Chotikapanich (1993), ^e^ Pareto = Functional form implied by Pareto distribution.

**Table 5d. The income data of the other 20 countries are used to demonstrate the performance of Paul and Shankar (2020)’s functional form and those of the other popular functional forms based on the estimated Gini index.** The bold numbers (if any) indicate that Paul and Shankar (2020)’s functional form is superior to the other existing widely used functional forms.

**Country	Year	Income share**

**of the top 20%**

**Observed Gini index	Estimated Gini index**

**PS**^a^	**KP**^b^	**A**^c^	**C**^d^	**Pareto**^e^

| Thailand | 2017 | 51.12 | 0.4528 | 0.4669 | 0.4507 | 0.4469 | 0.4496 | 0.4390 |
| --- | --- | --- | --- | --- | --- | --- | --- | --- |
| Malawi | 2017 | 51.67 | 0.4470 | 0.4600 | 0.4391 | 0.4404 | 0.4385 | 0.4345 |
| Ecuador | 2015 | 51.90 | 0.4620 | 0.4754 | 0.4594 | 0.4554 | 0.4582 | **0.4475** |
| Benin | 2015 | 52.13 | 0.4780 | 0.4842 | **0.4700** | **0.4656** | **0.4687** | **0.4585** |
| Philippines | 2012 | 52.68 | 0.4649 | 0.4812 | 0.4649 | 0.4607 | 0.4636 | 0.4526 |
| Sri Lanka | 2010 | 54.40 | 0.4900 | 0.5020 | 0.4865 | 0.4828 | 0.4850 | **0.4758** |
| Nicaragua | 2014 | 54.50 | 0.4950 | 0.5045 | 0.4898 | **0.4853** | 0.4883 | **0.4781** |
| Bangladesh | 2016 | 54.75 | 0.4980 | 0.5045 | **0.4891** | **0.4869** | **0.4877** | **0.4805** |
| Costa Rica | 2016 | 54.90 | 0.5000 | 0.5112 | 0.4982 | 0.4910 | 0.4965 | **0.4828** |
| Saint Lucia | 2016 | 55.39 | 0.5123 | 0.5191 | 0.5074 | **0.4994** | 0.5056 | **0.4915** |
| Paraguay | 2016 | 55.41 | 0.5100 | 0.5185 | 0.5058 | **0.4988** | 0.5040 | **0.4911** |
| Mexico | 2016 | 55.70 | 0.5040 | 0.5151 | 0.5001 | 0.4955 | 0.4984 | **0.4882** |
| India | 2012 | 56.38 | 0.5150 | 0.5262 | 0.5135 | 0.5063 | 0.5116 | **0.4986** |
| Eswatini | 2010 | 56.73 | 0.5145 | 0.5294 | 0.5167 | 0.5090 | 0.5147 | 0.5011 |
| Colombia | 2015 | 56.80 | 0.5240 | 0.5342 | 0.5222 | 0.5145 | 0.5202 | **0.5071** |
| Brazil | 2018 | 58.60 | 0.5240 | 0.5495 | 0.5380 | 0.5301 | 0.5358 | 0.5231 |
| Egypt | 2013 | 58.78 | 0.5400 | 0.5505 | 0.5378 | 0.5313 | 0.5356 | **0.5245** |
| Mozambique | 2015 | 59.53 | 0.5400 | 0.5545 | 0.5405 | 0.5352 | 0.5382 | 0.5283 |
| Honduras | 2012 | 60.10 | 0.5520 | 0.5633 | 0.5515 | 0.5437 | 0.5490 | **0.5369** |
| Namibia | 2016 | 63.74 | 0.5907 | 0.6052 | 0.5983 | 0.5846 | 0.5953 | 0.5791 |

Note: ^a^ PS = Paul and Shankar (2020), ^b^ KP = Kakwani and Podder (1973), ^c^ A = Aggarwal (1984), ^d^ C= Chotikapanich (1993), ^e^ Pareto = Functional form implied by Pareto distribution.

The overall results in Table 5a – 5d show that Paul and Shankar (2020)’s functional form is, by and large, outperformed by the other existing widely used functional forms, namely, Kakwani and Podder (1973), Aggarwal (1984), Chotikapanich (1993), and a functional form implied by Pareto distribution. The results in Table 5a show that, on the basis of R^2^, there are *only* 2 out of 100 cases where Paul and Shankar (2020)’s functional form performs better than the other existing widely used functional forms. Recall that Paul and Shankar (2020) show that, on the criteria of MSE and IIM, their functional form fits the income data of Australia better than the other existing widely used functional forms considered in their study. However, using the income data of the other 20 countries and the same performance assessment criteria as those used in Paul and Shankar (2020)’s study, the results in Table 5b and 5c indicate that their functional form is superior to the other existing widely used functional forms in *only* 9 out of 100 cases based on MSE and *only* 2 out of 100 cases based on IIM, respectively. Lastly, on the criterion of the estimated Gini index as measured by the absolute value of the difference between the estimated Gini index and the observed Gini index, the results in Table 5d show that the functional form proposed by Paul and Shankar (2020) outperforms the other popular functional specifications in 22 out of 100 cases. Fig. 2 illustrates the performance comparison between Paul and Shankar (2020)’s functional form and the other existing widely used functional forms considered in their study when the income data has a long-tailed property.

## Fig. 2. The performance comparison between Paul and Shankar (2020)’s functional form and the other existing widely used functional forms, namely, Kakwani and Podder (1973), Aggarwal (1984), Chotikapanich (1993), and functional form implied by Pareto distribution when the income data has a long-tailed property. (a) R^2^. (b) MSE. (c) IIM. (d) Estimated Gini index.

Although one of the aims to develop a functional form for the Lorenz curve is to compute inequality measures such as the Gini index that would be close to the actual observation (Paul and Shankar, 2020), we would like to note that the shape of income distribution is also important and should be taken into consideration when evaluating the performance of different functional forms. This is because it is possible that different functional forms could have the estimated Gini index that is identical to its observed value but the shapes of distributions are different. By using the income data of Thailand, this point could be demonstrated by finding the value of the estimated parameter of functional form proposed by Paul and Shankar (2020) and those of the

other popular functional forms that result in the same value of the estimated Gini index. The results are reported in Table 6.

## Table 6. The income data of Thailand are used to demonstrate the performance of Paul and Shankar (2020)’s functional form and those of the other popular functional forms all of which share the same value of the estimated Gini index which is equal to 0.4528.

| **PS**^a^ | | **KP**^b^ **A**^c^ **C**^d^ **Pareto**^e^ | | | |
| --- | --- | --- | --- | --- | --- |
| **Estimated parameter** | 𝛾 = 0.2273 | 𝛿 = 3.5401 | 𝜃 = 0.3482 | 𝑘 = 3.1328 | 𝛼 = 2.6547 |

**R^2^**	0.9797	0.9875	0.9977	0.9899	0.9882

| **MSE** | 0.0014 | 0.0010 | 0.0003 | 0.0009 | 0.0008 |
| --- | --- | --- | --- | --- | --- |
| **IIM** | 0.0498 | 0.0281 | 0.0083 | 0.0236 | 0.0260 |
| **Estimated Gini index** | 0.4528 | 0.4528 | 0.4528 | 0.4528 | 0.4528 |
| **Observed Gini index** |  |  | 0.4528 |  |  |

Note: ^a^ PS = Paul and Shankar (2020), ^b^ KP = Kakwani and Podder (1973), ^c^ A = Aggarwal (1984), ^d^ C= Chotikapanich (1993), ^e^ Pareto = Functional form implied by Pareto distribution.

The results in Table 6 show that the estimated parameters of all functional forms are different but all of them result in the same estimated Gini index which is 0.4528. Fig. 3 illustrates the actual Lorenz plot and the estimated Lorenz curves using Paul and Shankar (2020)’s functional form and the other existing widely used functional forms all of which share the same estimated Gini index.

## Fig. 3. The actual Lorenz plot and the estimated Lorenz curves using different functional forms all of which share the same value of the estimated Gini index which is equal to 0.4528.

Given that there are infinite number of the Lorenz curves that could yield the same value of the Gini index, a good functional form should be able to describe the shape of income distributions through the changes in the value of parameters and the fact that it fits the actual data would be the main reason for its choice (Dagum, 1977). Therefore, the shape of income distribution is relatively more important than how close the estimated Gini index is to its actual observation and the priority should be given to the values of goodness-of-fit statistics when assessing the performance of different functional forms. Viewed this way, the performance of

Paul and Shankar (2020)’s functional form compared to the other existing widely used functional forms considered in their study on the basis of the estimated Gini index, is at best mixed. It does not rank second closely behind that of Aggarwal (1984) as for the case when using the income data of Australia reported in their study.

### 3.4. The performance comparison on the basis of the estimated Gini index between single- parameter functional forms and a functional form that contains more than one parameter

Although the key advantage of any single-parameter functional form is its parsimony, we would like to point out that, in order to evaluate the performance of a functional form for the Lorenz curve on the criteria of the estimated Gini index, besides the issues of the shape of income distribution and the goodness-of-fit statistics as discussed above, a functional form that contains more than one parameter is required since the curvature of the estimated Lorenz curve has to be adjustable so that it would fit the actual observations as much as possible while keeping the value of the estimated Gini index constant. As noted by Dagum (1977), this cannot be done by using a single-parameter functional form since the estimated Gini index would be a monotonic function of it.

In order to illustrate that a functional form that contains more than one parameter could generally be used to compute the value of the estimated Gini index that would be closer to the actual observations than single-parameter functional forms, this study employs a two-parameter functional form proposed by Sitthiyot and Holasut (2021) to estimate the Lorenz curve using the same set of data on decile income shares of 40 countries. The reason that we choose Sitthiyot and Holasut (2021)’s functional specification is not only because their specified functional form has several advantages over the functional form proposed by Kakwani (1980) which, according to Cheong (2002) and Tanak et al. (2018), has the best overall performance among all popular

functional forms for the Lorenz curves but also has an explicit mathematical solution for the Gini index. By using the same notations for the cumulative normalized rank of income (𝑝) and the cumulative normalized income 𝐿(𝑝) as denoted in Materials and Methods and also let 𝑎 and 𝑏 be parameters, the specified functional form for the Lorenz curve proposed by Sitthiyot and Holasut (2021) is as follows:

1

(𝑝) = (1 − 𝑎) ∗ 𝑝^𝑏^ + 𝑎 ∗ (1 − (1 − 𝑝)𝑏),	(10)

0 ≤ 𝑎 ≤ 1,

1 ≤ 𝑏

This specified functional form satisfies all necessary and sufficient conditions for the Lorenz curve which are: 𝐿 (0) = 0, 𝐿 (1) = 1, (𝑝) is convex, ^𝑑𝐿^ ≥ 0, and ^𝑑^2𝐿 ≥ 0.

𝑑𝑝

𝑑𝑝^2^

According to Sitthiyot and Holasut (2021), the parameter *b* represents the degree of inequality in income distribution as measured by the Gini index. The parameter 𝑎 is the weight that controls the curvature of the Lorenz curve such that the Gini index remains constant since, for a given value of parameter 𝑏, there are infinite values of parameter 𝑎 that could give an identical value of the Gini index. The parameter 𝑎 thus provides the information about countries’ income shares in case their Lorenz curves intersect. In addition, the shape of the estimated Lorenz curve could be conveniently adjusted through the change in parameter 𝑎 while the value of the Gini index is kept constant. The values of parameters 𝑎 and 𝑏 estimated using Sitthiyot and Holasut (2021)’s functional form for the Lorenz curve are reported in Tables A1 and A2 in Appendix. The estimated Lorenz curves of 40 countries are then used to calculate the values of the estimated Gini index which in turn would be compared to those computed using single-parameter

functional forms considered in Paul and Shankar (2020)’s study. Tables 7a and 7b report our results.

**Table 7a. The income data of 20 OECD countries are used to demonstrate the performance of single-parameter functional forms and that of a two-parameter functional form proposed by Sitthiyot and Holasut (2021) on the criterion of estimated Gini index.** The bold numbers (if any) indicate that the single-parameter functional forms are superior to the two- parameter function form.

**Country	Year	Observed Gini index	Estimated Gini index**

**PS**^a^	**KP**^b^	**A**^c^	**C**^d^	**Pareto**^e^	**SH**^f^

| Slovakia | 2017 | 0.2320 | 0.4180 | 0.2242 | 0.2227 | 0.2239 | 0.2171 | 0.2300 |
| --- | --- | --- | --- | --- | --- | --- | --- | --- |
| Slovenia | 2017 | 0.2370 | 0.4180 | 0.2315 | 0.2305 | 0.2313 | 0.2250 | 0.2372 |
| Czech Republic | 2017 | 0.2450 | 0.4180 | 0.2386 | 0.2389 | 0.2387 | 0.2351 | 0.2445 |
| Finland | 2017 | 0.2530 | 0.4180 | 0.2470 | 0.2470 | 0.2470 | 0.2426 | 0.2530 |
| Belgium | 2017 | 0.2600 | 0.4180 | 0.2540 | 0.2526 | 0.2538 | 0.2463 | 0.2602 |
| Norway | 2017 | 0.2610 | 0.4180 | 0.2520 | 0.2514 | 0.2518 | 0.2467 | 0.2583 |
| Netherlands | 2017 | 0.2710 | 0.4180 | 0.2628 | 0.2622 | 0.2627 | 0.2568 | 0.2690 |
| Denmark | 2017 | 0.2760 | 0.4180 | 0.2646 | 0.2644 | 0.2646 | 0.2599 | 0.2711 |
| Austria | 2017 | 0.2790 | 0.4180 | 0.2711 | 0.2701 | 0.2709 | 0.2644 | 0.2776 |
| Sweden | 2017 | 0.2800 | 0.4180 | 0.2709 | 0.2697 | 0.2706 | 0.2639 | 0.2773 |
| Hungary | 2017 | 0.2810 | 0.4180 | 0.2739 | 0.2728 | 0.2737 | 0.2669 | 0.2802 |
| Germany | 2017 | 0.2910 | 0.4180 | 0.2833 | 0.2824 | 0.2831 | 0.2764 | 0.2898 |
| Poland | 2017 | 0.2920 | 0.4180 | 0.2843 | 0.2835 | 0.2842 | 0.2777 | 0.2907 |
| France | 2017 | 0.2930 | 0.4180 | 0.2834 | 0.2837 | 0.2834 | 0.2794 | 0.2899 |
| Switzerland | 2017 | 0.3010 | 0.4180 | 0.2927 | 0.2924 | 0.2926 | 0.2871 | 0.2992 |
| Ireland | 2017 | 0.3060 | 0.4180 | 0.3002 | 0.2995 | 0.3001 | 0.2933 | 0.3065 |
| Canada | 2017 | 0.3090 | 0.4180 | 0.3027 | 0.3004 | 0.3022 | 0.2929 | 0.3093 |
| Luxembourg | 2017 | 0.3090 | 0.4180 | 0.3037 | 0.3019 | 0.3034 | 0.2948 | 0.3102 |
| Estonia | 2017 | 0.3160 | 0.4180 | 0.3120 | 0.3087 | 0.3114 | 0.2999 | 0.3187 |
| Italy | 2017 | 0.3270 | 0.4180 | 0.3183 | 0.3156 | 0.3178 | 0.3079 | 0.3251 |

Note: ^a^ PS = Paul and Shankar (2020), ^b^ KP = Kakwani and Podder (1973), ^c^ A = Aggarwal (1984), ^d^ C= Chotikapanich (1993), ^e^ Pareto = Functional form implied by Pareto distribution, ^f^ SH = Sitthiyot and Holasut (2021).

**Table 7b. The income data of the other 20 countries are used to demonstrate the performance of single-parameter functional forms and that of a two-parameter functional form proposed by Sitthiyot and Holasut (2021) on the criterion of estimated Gini index.** The bold numbers (if any) indicate that the single-parameter functional forms are superior to the two- parameter function form.

**Country	Year	Observed Gini index	Estimated Gini index**

**PS**^a^	**KP**^b^	**A**^c^	**C**^d^	**Pareto**^e^	**SH**^f^

| Thailand | 2017 | 0.4528 | 0.4669 | **0.4507** | 0.4469 | 0.4496 | 0.4390 | 0.4548 |
| --- | --- | --- | --- | --- | --- | --- | --- | --- |
| Malawi | 2017 | 0.4470 | 0.4600 | 0.4391 | 0.4404 | 0.4385 | 0.4345 | 0.4425 |
| Ecuador | 2015 | 0.4620 | 0.4754 | 0.4594 | 0.4554 | 0.4582 | 0.4475 | 0.4632 |
| Benin | 2015 | 0.4780 | 0.4842 | 0.4700 | 0.4656 | 0.4687 | 0.4585 | 0.4742 |
| Philippines | 2012 | 0.4649 | 0.4812 | **0.4649** | 0.4607 | **0.4636** | 0.4526 | 0.4680 |
| Sri Lanka | 2010 | 0.4900 | 0.5020 | 0.4865 | 0.4828 | 0.4850 | 0.4758 | 0.4889 |
| Nicaragua | 2014 | 0.4950 | 0.5045 | 0.4898 | 0.4853 | 0.4883 | 0.4781 | 0.4924 |
| Bangladesh | 2016 | 0.4980 | **0.5045** | 0.4891 | 0.4869 | 0.4877 | 0.4805 | 0.4913 |
| Costa Rica | 2016 | 0.5000 | 0.5112 | 0.4982 | 0.4910 | 0.4965 | 0.4828 | 0.5008 |
| Saint Lucia | 2016 | 0.5123 | 0.5191 | 0.5074 | 0.4994 | 0.5056 | 0.4915 | 0.5100 |
| Paraguay | 2016 | 0.5100 | 0.5185 | 0.5058 | 0.4988 | 0.5040 | 0.4911 | 0.5080 |
| Mexico | 2016 | 0.5040 | 0.5151 | 0.5001 | 0.4955 | 0.4984 | 0.4882 | 0.5014 |
| India | 2012 | 0.5150 | 0.5262 | 0.5135 | 0.5063 | 0.5116 | 0.4986 | 0.5149 |
| Eswatini | 2010 | 0.5145 | 0.5294 | **0.5167** | 0.5090 | **0.5147** | 0.5011 | 0.5178 |
| Colombia | 2015 | 0.5240 | 0.5342 | 0.5222 | 0.5145 | 0.5202 | 0.5071 | 0.5235 |
| Brazil | 2018 | 0.5240 | 0.5495 | 0.5380 | **0.5301** | **0.5358** | **0.5231** | 0.5379 |
| Egypt | 2013 | 0.5400 | 0.5505 | **0.5378** | 0.5313 | 0.5356 | 0.5245 | 0.5370 |
| Mozambique | 2015 | 0.5400 | 0.5545 | **0.5405** | 0.5352 | 0.5382 | 0.5283 | 0.5383 |
| Honduras | 2012 | 0.5520 | 0.5633 | **0.5515** | 0.5437 | 0.5490 | 0.5369 | 0.5495 |
| Namibia | 2016 | 0.5907 | 0.6052 | 0.5983 | 0.5846 | 0.5953 | 0.5791 | 0.5943 |

Note: ^a^ PS = Paul and Shankar (2020), ^b^ KP = Kakwani and Podder (1973), ^c^ A = Aggarwal (1984), ^d^ C= Chotikapanich (1993), ^e^ Pareto = Functional form implied by Pareto distribution, ^f^ SH = Sitthiyot and Holasut (2021).

The results on the estimated Gini index shown in Tables 7a and 7b indicate that the two- parameter functional form proposed by Sitthiyot and Holasut (2021), by and large, outperforms single-parameter functional forms used in Paul and Shankar (2020)’s study. As shown in both Tables 7a and 7b, there are *only* 12 cases out of 200 cases where the single-parameter functional forms give the values of the estimated Gini index that are closer to their actual observations than the two-parameter functional form proposed by Sitthiyot and Holasut (2021), all of which are from countries whose upper part of the income distribution has a long-tailed property. These results confirm that the two-parameter functional form, in general, performs better than one- parameter functional forms on the criterion of the estimated Gini index.

## 4. Conclusions and remarks

Paul and Shankar (2020) propose an alternative single-parameter functional form for the Lorenz curve and use the Australian income data between 2001 and 2010 that have the Gini index between 0.4442 and 0.4633 to show that their functional form is superior to the other existing widely used functional forms. Given that previous studies pointed out that an excellent performance of any parametric functional form for the Lorenz curve that is based on a single country case study and a limited range of distribution must be treated with extreme caution (Cowell and Mehta, 1982; Shorrocks and Wan, 2008; Jorda et al., 2021), this study demonstrates that when the observed Gini index is lower than the critical threshold which is found to be 0.4180 as for the cases of 20 OECD countries, the functional form proposed by Paul and Shankar (2020) not only has a serious limitation in that it fails to fit the actual observations well but also is outperformed by the other existing widely used functional forms. This study also shows that when the upper part of income distribution has a long-tailed property as for the cases of the other

20 different countries, the functional form proposed by Paul and Shankar (2020) is, by and large, outperformed by the other existing widely used functional forms considered in their study.

This study would like to remark that, for future evaluation of any single parametric functional form for the Lorenz curve, the goodness-of-fit statistics should be given the priority since the shape of income distribution is more important than the estimated Gini index. As noted by Dagum (1977), whenever a functional form contains a single inequality parameter, the estimated Gini index would be a monotonic function of it, and hence, it would not be able to detect the intersecting Lorenz curves. To evaluate the performance on the criterion of the estimated Gini index, a functional form that contains more than one parameter is required since the curvature of the estimated Lorenz curve has to be adjustable so that it would fit the actual observations as much as possible while keeping the value of the estimated Gini index the same. To illustrate this point, this study employs the two-parameter functional form developed by Sitthiyot and Holasut (2021) to estimate the Lorenz curve and compute the value of the estimated Gini index using the grouped data on income shares of 40 countries. The overall results indicate that the two-parameter functional form, by and large, performs better than the single-parameter functional forms considered in Paul and Shankar (2020)’s study on the criterion of the value of the estimated Gini index.

Last but not least, before using any functional form to estimate the Lorenz curve, policymakers should be aware that the goodness-of-fit statistical measures, the shape of the estimated Lorenz curve, and the estimated Gini index should be taken into consideration altogether. The key lesson learned from Paul and Shankar (2020)’s study is that their results should be treated with great caution since all of them rely on a single country case study and a narrow range of distribution (Cowell and Mehta, 1982; Shorrocks and Wan, 2008; Jorda et al.,

2021). As noted by Dagum (1977), a good functional form for estimating the Lorenz curve must describe the shape of income distributions of different countries, regions, socioeconomic groups, and in different time periods through the changes in parameter values. If the choice is not a valid candidate for representing the income distribution, the estimates on the income shares and inequality measures might be severely affected by misspecification bias (Jorda et al., 2021).

Thus, before applying any functional form for estimating the Lorenz curve, policymakers should carefully check whether or not it satisfies the aforementioned criteria suggested by Dagum (1977). This is because using a functional form that does not fit actual observations could adversely affect inequality measures and income distribution policies.

## Appendix

**Table A1. The values of parameters estimated based on Paul and Shankar (2020)’s functional form and the other existing widely used single-parameter functional forms as well as the functional form proposed by Sitthiyot and Holasut (2021) using the income data of 20 OECD countries.**

**Country	Year	Observed Gini index**

**Estimated parameter	PS**^a^	**KP**^b^	**A**^c^	**C**^d^	**Pareto**^e^	**SH**^f^

|  | | | 𝛾 | 𝛿 | 𝜃 | 𝑘 | 𝛼 | 𝑎 | 𝑏 |
| --- | --- | --- | --- | --- | --- | --- | --- | --- | --- |
| Slovakia | 2017 | 0.2320 | 0.0000001 | 0.8147 | 0.1680 | 1.3856 | 1.5545 | 0.3971 | 1.5974 |
| Slovenia | 2017 | 0.2370 | 0.0000001 | 0.8473 | 0.1739 | 1.4346 | 1.5808 | 0.4639 | 1.6221 |
| Czech Republic | 2017 | 0.2450 | 0.0000001 | 0.8798 | 0.1804 | 1.4840 | 1.6146 | 0.6009 | 1.6473 |
| Finland | 2017 | 0.2530 | 0.0000001 | 0.9183 | 0.1865 | 1.5396 | 1.6407 | 0.5800 | 1.6772 |
| Belgium | 2017 | 0.2600 | 0.0000001 | 0.9513 | 0.1908 | 1.5852 | 1.6536 | 0.4484 | 1.7034 |
| Norway | 2017 | 0.2610 | 0.0000001 | 0.9415 | 0.1899 | 1.5722 | 1.6549 | 0.5214 | 1.6965 |
| Netherlands | 2017 | 0.2710 | 0.0000001 | 0.9930 | 0.1982 | 1.6458 | 1.6912 | 0.5302 | 1.7361 |
| Denmark | 2017 | 0.2760 | 0.0000001 | 1.0019 | 0.1999 | 1.6588 | 1.7022 | 0.5649 | 1.7438 |
| Austria | 2017 | 0.2790 | 0.0000001 | 1.0335 | 0.2043 | 1.7025 | 1.7187 | 0.5028 | 1.7684 |
| Sweden | 2017 | 0.2800 | 0.0000001 | 1.0322 | 0.2040 | 1.7005 | 1.7171 | 0.4928 | 1.7676 |
| Hungary | 2017 | 0.2810 | 0.0000001 | 1.0469 | 0.2064 | 1.7213 | 1.7280 | 0.5052 | 1.7786 |
| Germany | 2017 | 0.2910 | 0.0000001 | 1.0941 | 0.2137 | 1.7872 | 1.7641 | 0.5236 | 1.8160 |
| Poland | 2017 | 0.2920 | 0.0000001 | 1.0991 | 0.2146 | 1.7944 | 1.7690 | 0.5347 | 1.8199 |
| France | 2017 | 0.2930 | 0.0000001 | 1.0942 | 0.2148 | 1.7891 | 1.7754 | 0.6223 | 1.8165 |
| Switzerland | 2017 | 0.3010 | 0.0000001 | 1.1417 | 0.2215 | 1.8537 | 1.8055 | 0.5805 | 1.8539 |
| Ireland | 2017 | 0.3060 | 0.0000001 | 1.1807 | 0.2270 | 1.9067 | 1.8299 | 0.5604 | 1.8841 |
| Canada | 2017 | 0.3090 | 0.0000001 | 1.1937 | 0.2277 | 1.9224 | 1.8285 | 0.4488 | 1.8958 |
| Luxembourg | 2017 | 0.3090 | 0.0000001 | 1.1990 | 0.2288 | 1.9303 | 1.8359 | 0.4872 | 1.8995 |
| Estonia | 2017 | 0.3160 | 0.0000001 | 1.2433 | 0.2341 | 1.9882 | 1.8566 | 0.3955 | 1.9355 |
| Italy | 2017 | 0.3270 | 0.0000001 | 1.2775 | 0.2395 | 2.0346 | 1.8898 | 0.4382 | 1.9636 |

Note: ^a^ PS = Paul and Shankar (2020), ^b^ KP = Kakwani and Podder (1973), ^c^ A = Aggarwal (1984), ^d^ C= Chotikapanich (1993), ^e^ Pareto = Functional form implied by Pareto distribution, ^f^ SH = Sitthiyot and Holasut (2021).

## Table A2. The values of parameters estimated based on Paul and Shankar (2020)’s functional form and the other existing widely used single-parameter functional forms as well as the functional form proposed by Sitthiyot and Holasut (2021) using the income data of the other 20 countries.

**Country	Year	Income share of**

**Estimated parameter	PS**^a^	**KP**^b^	**A**^c^	**C**^d^	**Pareto**^e^	**SH**^f^

**the top 20%**

|  |  |  | 𝛾 | 𝛿 | 𝜃 | 𝑘 | 𝛼 | 𝑎 | 𝑏 |
| --- | --- | --- | --- | --- | --- | --- | --- | --- | --- |
| Thailand | 2017 | 51.12 | 0.3202 | 2.1398 | 0.3434 | 3.1035 | 2.5652 | 0.6113 | 2.6686 |
| Malawi | 2017 | 51.67 | 0.2748 | 2.0508 | 0.3381 | 3.0030 | 2.5370 | 0.8016 | 2.5874 |
| Ecuador | 2015 | 51.90 | 0.3759 | 2.2089 | 0.3503 | 3.1833 | 2.6197 | 0.6174 | 2.7255 |
| Benin | 2015 | 52.13 | 0.4340 | 2.2953 | 0.3586 | 3.2827 | 2.6935 | 0.5923 | 2.8036 |
| Philippines | 2012 | 52.68 | 0.4143 | 2.2532 | 0.3546 | 3.2340 | 2.6534 | 0.6332 | 2.7597 |
| Sri Lanka | 2010 | 54.40 | 0.5525 | 2.4359 | 0.3727 | 3.4418 | 2.8154 | 0.6746 | 2.9134 |
| Nicaragua | 2014 | 54.50 | 0.5695 | 2.4653 | 0.3747 | 3.4745 | 2.8318 | 0.6459 | 2.9397 |
| Bangladesh | 2016 | 54.75 | 0.5695 | 2.4653 | 0.3747 | 3.4745 | 2.8318 | 0.7263 | 2.9313 |
| Costa Rica | 2016 | 54.90 | 0.6142 | 2.5408 | 0.3794 | 3.5572 | 2.8667 | 0.5704 | 3.0066 |
| Saint Lucia | 2016 | 55.39 | 0.6680 | 2.6257 | 0.3863 | 3.6514 | 2.9332 | 0.5480 | 3.0815 |
| Paraguay | 2016 | 55.41 | 0.6639 | 2.6105 | 0.3858 | 3.6347 | 2.9301 | 0.5906 | 3.0648 |
| Mexico | 2016 | 55.70 | 0.6410 | 2.5575 | 0.3832 | 3.5767 | 2.9078 | 0.6823 | 3.0113 |
| India | 2012 | 56.38 | 0.7166 | 2.6837 | 0.3920 | 3.7149 | 2.9887 | 0.6088 | 3.1230 |
| Eswatini | 2010 | 56.73 | 0.7382 | 2.7149 | 0.3943 | 3.7485 | 3.0087 | 0.6073 | 3.1479 |
| Colombia | 2015 | 56.80 | 0.7711 | 2.7691 | 0.3988 | 3.8083 | 3.0576 | 0.6016 | 3.1969 |
| Brazil | 2018 | 58.60 | 0.8784 | 2.9303 | 0.4119 | 3.9823 | 3.1939 | 0.6362 | 3.3279 |
| Egypt | 2013 | 58.78 | 0.8854 | 2.9290 | 0.4129 | 3.9807 | 3.2057 | 0.6917 | 3.3196 |
| Mozambique | 2015 | 59.53 | 0.9134 | 2.9572 | 0.4162 | 4.0101 | 3.2401 | 0.7599 | 3.3314 |
| Honduras | 2012 | 60.10 | 0.9763 | 3.0767 | 0.4234 | 4.1373 | 3.3190 | 0.6923 | 3.4399 |
| Namibia | 2016 | 63.74 | 1.2904 | 3.6505 | 0.4584 | 4.7346 | 3.7517 | 0.6163 | 3.9296 |

Note: ^a^ PS = Paul and Shankar (2020), ^b^ KP = Kakwani and Podder (1973), ^c^ A = Aggarwal (1984), ^d^ C= Chotikapanich (1993), ^e^ Pareto = Functional form implied by Pareto distribution, ^f^ SH = Sitthiyot and Holasut (2021).

## Author Contributions:

Conceptualization: Thitithep Sitthiyot; Methodology: Thitithep Sitthiyot, Kanyarat Holasut; Formal analysis: Thitithep Sitthiyot; Validation: Kanyarat Holasut; Writing – original draft: Thitithep Sitthiyot; Writing – review and editing: Thitithep Sitthiyot, Kanyarat Holasut.

## References

Aggarwal, V., 1984. On optimum aggregation of income distribution data. Sankhya B. 46, 343- 355.

Cheong, K. S., 2002. An empirical comparison of alternative functional forms for the Lorenz curve. Appl. Econ. Lett. 9, 171-176.

Chotikapanich, D., 1993. A comparison of alternative functional forms for the Lorenz curve. Econ. Lett. 41, 129-138.

Cowell, F. A., Mehta, F., 1982. The estimation and interpolation of inequality measures. Rev. Econ. Stud. 49, 273-290.

Dagum, C., 1977. A new model of personal income distribution: Specification and estimation. In: Chotikapanich, D. (Ed.), Modeling income distributions and Lorenz curves. Economic studies in equality, social exclusion and well-being, vol 5. Springer, New York, pp. 3-25. https://doi.org/10.1007/978-0-387-72796-7_1

Jorda, V., Sarabia, J. M., Jäntti, M., 2021. Inequality measurement with grouped data: Parametric and non-parametric methods. J. R. Stat. Soc. Series A. 184, 964-984. <https://doi.org/10.1111/rssa.12702>

Kakwani, N. C., 1980. On a Class of Poverty Measures. Econometrica. 48, 437-446.

Kakwani, N. C., Podder, N., 1973. On the estimation of Lorenz curves from grouped observations. Int. Econ. Rev. 14, 278-292.

Paul, S., Shankar, S., 2020. An alternative single parameter functional form for Lorenz curve. Empir. Econ. 59, 1393-1402. <https://doi.org/10.1007/s00181-019-01715-3>

Shorrocks, A. F., Wan, G., 2008. Ungrouping income distributions: Synthesising samples for inequality and poverty analysis. Research Paper No. 2008/16, United Nations University World Institute for Development Economics Research (UNU-WIDER). https:/[/www.wider.unu.edu/publication/ungroupin](http://www.wider.unu.edu/publication/ungrouping-income-distributions)g[-income-distributions](http://www.wider.unu.edu/publication/ungrouping-income-distributions)

Sitthiyot, T., Holasut, K., 2021. A simple method for estimating the Lorenz curve. Humanit. Soc. Sci. Commun. 8, 268. https://doi.org/10.1057/s41599-021-00948-x

Tanak, A. K., Mohtashami Borzadaran, G. R., Ahmadi, J., 2018. New functional forms of Lorenz curves by maximizing Tsallis entropy of income share function under the constraint on generalized Gini index. Physica A. 511, 280-288.

[dataset]. The Office of the National Economic and Social Development Council. 2021. Poverty and Income Distribution Statistics. https:/[/w](http://www.nesdc.go.th/main.php?filename=social)w[w.nesdc.go.th/main.php?filename=social](http://www.nesdc.go.th/main.php?filename=social)

[dataset]. The United Nations University World Institute for Development Economics Research (UNU-WIDER). 2020. World Income Inequality Database (WIID), Version date: 6 May 2020. https://www4.wider.unu.edu/

[dataset]. The World Bank. 2020. World Bank Open Data. https://data.worldbank.org/
